# Frequency of alcohol use and obesity in community medicine patients

**DOI:** 10.1186/1471-2296-6-17

**Published:** 2005-04-22

**Authors:** James E Rohrer, Barbara M Rohland, Anne Denison, Anthony Way

**Affiliations:** 1 Health Services Research, Department of Family and Community Medicine, Texas Tech University Health Sciences Center, Amarillo, Texas, United States; 2 Department of Psychiatry, Texas Tech University Health Sciences Center, Amarillo, Texas, USA; 3 Preventive Medicine, Department of Family and Community Medicine, Texas Tech University Health Sciences Center, Lubbock, Texas, USA

## Abstract

**Background:**

Obesity is an important public health problem. However, the effects of alcohol use on the risk for obesity have not been thoroughly explored. This study focuses on how frequency of alcohol use is related to the risk of obesity in a community medicine clinic population.

**Methods:**

This study used a cross-sectional survey to test the hypothesis that obesity (BMI > 30) is associated with alcohol use. The convenience sample was drawn from three clinics that primarily serve low-income populations. Independent variables included frequency of alcohol use, frequency of binge drinking, demographic characteristics, health behaviors and health status.

**Results:**

In comparison to non-drinkers, people who consumed alcohol 3 or more days per month had lower odds of being obese (Adjusted Odds Ratio = .49, p < .04). As expected, there was a significant association between watching eight or more hours of television per day and obesity (AOR = 2.34, p < .01).

**Conclusion:**

More frequent drinking and less television time are independently associated with reduced odds of obesity in this sample of community medicine patients. Additional research is needed to isolate casual mechanisms.

## Background

Overweight and obesity are subjects of much public health scrutiny and concern. Obesity is significantly correlated with many chronic diseases, including diabetes, cardiovascular disease, some cancers, and gallbladder disease. The Centers for Disease Control and Prevention (CDC) estimates that in 2003 healthcare expenditures attributable to obesity reached $75 billion [1].

Weight control involves a complex interaction of physical, genetic, psycho-social and environmental factors. Addressing the problem requires a solid understanding of the risk factors and how they can be influenced. Epidemiologists have studied the health consequences of obesity [2-4], and several studies have explored the risk factors for obesity [5-13]. The health behaviors that determine obesity, such as eating too much and exercising too little, are related to other health behaviors such as cigarette smoking and alcohol consumption. However, no consensus has emerged as to casual connections among these behaviors.

Investigators from the National Institutes of Health analyzed the 2001 National Health Interview Survey to estimate the prevalence of multiple behavioral risk factors for chronic diseases [14]. These investigators found that 17 percent of adults had three or more of the following risk factors: cigarette smoking, risky drinking of alcoholic beverages, physical inactivity, and overweight. Less than ten percent of Americans had zero risk factors. About 73 percent had only one or two risk factors, proving that unhealthy behaviors do not always co-occur. We note that smoking illustrates the complexity of these relationships since it is a risk factor for most chronic diseases but is inversely related to obesity.

The purpose of this study was to investigate the importance of alcohol consumption in predicting self-reported obesity in a sample of primary care patients visiting community medicine clinics. The analysis adjusts for the impact of other health behaviors.

## Methods

This study used a cross-sectional survey to test the hypothesis that obesity (BMI > 30) is associated with alcohol consumption in a community medicine population. This study was approved by the Institutional Review Board of Texas Tech Health Sciences Center (Amarillo). The convenience sample was drawn from three clinics that serve low-income populations. Participation was voluntary. Pregnant women and persons under age 18 were excluded from participation. Of 1471 surveys distributed to patients and accompanying adult family members, completed forms were received from 793 (54%). Return rates varied by clinic. Height and weight responses were complete for 747 subjects.

Clinic 1 is a university-based family medicine clinic providing a full range of primary care services to cross-generational clients. It is staffed by family medicine physicians and residents. Daily census was approximately 85 clients daily, of which, less than 5 % were non-English speaking. Clinic personnel distributed 500 survey forms over an eight week period with an 80.8% return rate. Clinic 2 serves women and children, providing obstetrical, well care (including immunizations), and acute care services to a targeted high-risk, low socioeconomic sub-population. It is staffed by pediatric and OB-GYN physicians and residents. Approximately 30% of the clinic clients do not speak English. A total of 471 surveys were distributed over a period of 18 weeks with a return of 37.6%. Both the large number of obstetrical patients (ineligible for survey) and percentage of non-English speaking clients contributed to the low return rate. Clinic 3 provides primary care services to a population of indigent adults meeting residential and income screening requirements. It is staffed by internal medicine physicians and residents. A total of 500 surveys were distributed in this clinic over a ten week period with a return of 42.6%.

The dependent variable was BMI calculated from self-reported height and weight data. Of 793 completed surveys, a BMI could be calculated for 747. The continuous BMI variable was collapsed into two classifications: Non-Obese (BMI <30) and Obese (BMI ≥ 30).

Alcohol use was measured by both frequency and intensity. Frequency of alcohol consumption was measured as days per month (grouped into none, 1–2 and 3 or more). Intensity of alcohol consumption as measured by frequency of bingeing (times per month when drank five or more drinks, grouped as none, 1–2 and 3 or more). Other independent variables included gender, age, race/ethnicity (non-Hispanic White, Hispanic, Black, other), marital status (married vs single), educational level (less than high school, high school, some college, college graduate), number of persons living in the home (just me, one additional person, two or more), number of places to walk (none, one place, two or more), worry about having enough food (yes vs no), frequent mental distress (less than 15 days per month versus 15 or more), hours spent watching TV daily (less than 3, 3–7, or 8 or more), number of cigarettes per day (none, 1–20, more than 20), days per week of impaired activities of daily living (none vs one or more), days per week of exercise (none, 1–3, 4 or more) and health confidence. Health confidence was measured by the respondent's confidence that he or she can take of her own health (strongly agree, agree, not sure, disagree, strongly disagree).

Initial bivariate analysis using Pearson χ^2 ^was used to identify associations between obesity and the independent variables. Variables with *p *values less than 0.1 in the bivariate tests were retained for inclusion in a multivariate logistic regression model. Some cases were dropped due to missing data, resulting in a final sample of 594 in the regression model.

## Results

Of the 747 calculated BMI measurements, 515 (65%) fell into the overweight and obese categories. This is very similar to the BRFSS 2002 overweight/obese percentage of 63% for the state of Texas as a whole. Approximately, 40% of the sample BMI measurements were equal or greater than 30 (obese range). About 70% of respondents were non-drinkers and about 90% never binged.

In the univariate analyses, there was no statistically significant difference in obesity by gender, race/ethnicity, educational level, marital status or family size. Days per month of alcohol use was associated with obesity (p = .001), as was intensity (p = .01). Both bingers and daily drinkers were less likely to be obese. Age was significantly associated with obesity, as was number of places to walk, worry about having enough food, health confidence, frequent mental distress, hours watching TV, not smoking cigarettes, frequent mental distress, days with impaired ADLs, and days per week of exercise (see Tables 1 and 2).

**Table 1:** Percent Obese by Socioeconomic Characteristics of Sample (N = 747)

	**Overall**	**Not Obese**	**Obese**	***p*-value**
	% (#)	%	%	
**Gender%**				0.734
Male	19.46 (144)	61.11	38.89	
Female	80.45 (596)	59.56	40.44	
**Age group%**				0.004
Under 35	36.44 (270)	66.67	33.33	
35–45	19.57 (145)	56.55	43.45	
46–55	18.89 (140)	48.57	51.43	
56–65	12.42 (92)	56.52	43.48	
Over 65	12.69 (94)	65.96	34.04	
**Race/ethnicity%**				0.367
non-Hispanic White	67.40 (490)	61.22	38.78	
Hispanic	23.93 (174)	56.90	43.10	
Black	7.29 (53)	50.94	49.06	
Other	1.38 (10)	70.00	30.00	
**Marital status%**				0.145
Married	52.94 (234)	62.57	37.43	
Single	47.46 (140)	57.30	42.70	
**Educational status%**				0.148
< high school degree	8.67 (35)	54.69	45.31	
high school degree	36.86 (272)	64.71	35.29	
Some college	38.48 (284)	55.99	44.01	
college graduate	15.99 (118)	61.86	38.14	
**Number of persons living in the home%**				0.956
Just me	16.21 (119)	60.50	39.50	
One additional person	26.29 (193)	60.62	39.38	
Two or more	57.49 (422)	59.48	40.52	
**Number of places to walk%**				0.023
None	29.91 (210)	52.38	47.62	
One place	15.95 (112)	60.71	39.29	
Two or more	54.13 (380)	63.95	36.05	
**Worried about having enough food%**				0.033
Yes	26.91 (194)	53.09	46.91	
No	73.09 (527)	61.86	38.14	

**Table 2:** Percent Obese by Health and Behavioral Characteristics of Sample (N = 747)

	**Overall**	**Not Obese**	**Obese**	***p*-value**
	% (#)	%	%	
**Usually can take care of own health%**				0.020
Strongly agree	20.08 (142)	69.72	30.28	
Agree	35.79 (253)	62.06	37.94	
Not sure	13.30 (94)	53.19	46.81	
Disagree	22.21 (157)	52.87	47.13	
Strongly disagree	8.63 (61)	55.74	44.26	
**Frequent mental distress%**				0.001
Less than 15 days/month	72.50 (514)	63.81	36.19	
15 days or more	27.50 (195)	50.26	49.74	
**Days with impaired ADL%**				0.027
None	58.92 (416)	63.46	36.54	
One or more	41.08 (290)	55.17	44.83	
**Hours spent watching TV daily%**				0.001
Less than 3	39.41 (256)	65.63	34.38	
3 to 7 hours	50.00 (327)	54.74	45.26	
8 or more	10.86 (71)	42.25	57.75	
**Number of cigarettes per day%**				0.008
None	71.72 (535)	56.26	43.74	
1 to 20	20.11 (150)	68.67	31.33	
More than 20	8.18 (61)	68.85	31.15	
**Drink days per month%**				0.001
None	69.45 (516)	55.62	44.38	
1 to 2	16.15 (120)	65.83	34.17	
3 or more	14.14 (107)	73.83	26.17	
**5+ Drinks/day per month %**				0.010
None	86.35 (639)	58.22	41.78	
1 to 2	7.85 (58)	62.07	37.93	
3 or more	5.81 (43)	81.40	18.60	
**Days/Week Exercise%**				0.005
None	31.65 (226)	57.96	42.04	
1 to 3	45.10 (322)	54.04	45.96	
4 or more	23.25 (166)	69.28	30.72	

In the multivariate analysis, having places to walk, days with impaired ADLs, and being worried about food, and days of exercise per week were not found to be independently related to obesity (Table 3). Obesity was inversely related to cigarette smoking. Specifically, in comparison to non-smokers, persons who smoked 1–20 cigarettes per day had significantly lower adjusted odds of being obese (AOR = .56) and persons who smoked more than 20 cigarettes per day did as well (AOR = .33).

**Table 3:** Multiple logistic regression analysis* of obesity (BMI greater than or equal to 30) in community medicine patients (N = 594)

	**Odds Ratio (95% C.I.)**	***p*-value**
**Days/Week Exercise**		
None	Reference	
One to three	1.22 (0.81–1.86)	0.341
Four or more	0.61 (0.37–1.01)	0.054
**Usually can take care of own health**		
Strongly Agree	Reference	
Agree	1.32 (0.79–2.19)	0.286
Not Sure	2.01 (1.05–3.82)	0.034
Disagree	1.54 (0.88–2.71)	0.133
Strongly disagree	1.14 (0.54–2.41)	0.722
**Frequent mental distress**		
Less than 15 days	Reference	
15 days or more	1.56 (1.01–2.40)	0.043
**Hours spent watching TV daily**		
Less than 3	Reference	
3 to 7 hours	1.44 (0.97–2.12)	0.068
8 or more	2.34 (1.23–4.46)	0.009
**Number of cigarettes per day**		
None	Reference	
1 to 20	0.56 (0.35–0.89)	0.014
More than 20	0.33 (0.16–0.68)	0.003
**Drink days/month**		
None	Reference	
1 to 2	0.61 (0.35–1.05)	0.074
3 or more	0.49 (0.25–0.96)	0.037
**5+ Drinks/day per month**		
None	Reference	
1 to 2	1.13 (0.52–2.46)	0.764
3 or more	0.91 (0.33–2.54)	0.856

*Adjusted for age, places to walk, worried about having enough food, days with impaired activities of daily living.

In comparison to non-drinkers, people who consumed alcohol 3 or more days per month had lower odds of being obese (AOR = .49, p = .037). Binge drinking was not significantly related to obesity. However, we note that only 43 respondents binged three or more times per month. Therefore, power may not have been adequate to test this hypothesis.

As expected, there was a strong association between watching eight or more hours of television per day and obesity (AOR = 2.34, p<.01).

Persons who were not sure about the statement "I have usually been able to take care of my own health without the help of a doctor or a nurse" had increased odds of being obese (adjusted odds ratio = 2.01, p = 0.034). There was a higher degree of poor mental health as measured by frequent mental distress among obese respondents (AOR = 1.56, p = 0.043).

## Discussion

Recent epidemiological studies of alcohol use and health have measured alcohol use in a variety of ways. Some measured frequency of alcohol use while others measured intensity (drinks per occasion or total drinks) and some measure alcohol use both ways. Researchers also measured obesity in several different ways. These have included body mass index, waist circumference, and hip circumference.

The relationship between episodic heavy alcohol use and overall self-rated health was examined by Okosun et al [15]. Episodic heavy drinking was defined as consumption of 5 or more drinks at one occasion (for men) or 4 or more drinks (for women). This kind of bingeing was a significant risk factor for poor self-rated health. Okosun et al did not report any findings relating to total drinks per week or number of drinking episodes.

Paschall, Freisthler and Lipton, who studied alcohol use and depression among young adults created seven categories of alcohol use based on drinks per occasion rather than frequency of drinking [16]. They defined moderate drinking as no more than 1–2 drinks per occasion. Their analysis of data from the National Longitudinal Study of Adolescent Health showed that moderate drinkers were less likely to have depressive symptoms than either heavy drinkers or abstainers. Since depression and alcohol use both are related to obesity, this is an important finding.

Vadstrup et al studied waist circumference in a large population survey of Danish adults [10]. They measured alcohol use with total number of drinks per week but counted neither the number of drinking days nor the number of drinks per occasion. Vadstrup et al found the smallest waist circumference in persons who consumed 1–7 drinks per week.

In another Danish population-based study, Tolstrup et al studied both frequency of drinking and total alcohol intake [11]. They found obesity to be positively correlated with total drinks consumed but inversely correlated with frequency of drinking. Obesity was measured by waist circumference and hip circumference.

Finally, we note that Breslow and Smothers found a positive and linear relationship between BMI and quantity of alcohol, while drinking frequency was positively related to BMI [12]. The lowest BMI was found among persons who drank small quantities frequently. This study was limited to persons who never smoked, so the results may not be generalizable.

Our study differs from previous research in that it uses a sample drawn from a primary care population that is largely low-income. We employed BMI to measure obesity and we measured alcohol use with drinking frequency as well as intensity. Our measure of intensity (binge episodes) was answered too infrequently to allow for firm conclusions. With this design, we found that persons who drink more often were less likely to be obese. This finding corroborates the results reported by Breslow and Smothers. Since our study appears to be the first to focus on drinking frequency in a low-income primary care population, we think the results are useful to investigators who study the epidemiology of obesity.

Drinking frequency is inversely related to obesity in this sample of primary care patients who visited community medicine clinics. While it is unlikely that alcohol consumption has a direct beneficial effect on obesity, we suspect that a report of no alcohol consumption may be a proxy for some other, unknown variable that increases risk of obesity. One hypothesis is that persons who do not consume any alcohol may be following a religious prohibition that is associated with other behaviors that relate to obesity, such as group dining involving high-calorie meals. Another possible explanation is that a subgroup of persons who refrain from all alcohol but have frequent mental distress may be using food as a method for coping with stress.

Our finding about the relationship between drinking and lower risk of obesity clearly should be replicated in other studies before attributing it to any theory or generalizing it to other populations. Nevertheless, we note that earlier studies have reported that non-drinkers are at increased risk of obesity and the National Institute on Alcohol Abuse and Alcoholism (NIAAA) has found some benefits from moderate use of alcohol [8,13,14,17-19]. The NIAAA reported that the relationship between moderate alcohol consumption and weight gain, BMI, or obesity remains inconclusive. At the same time, there is some protective effect of moderate alcohol consumption on two major sequelae of obesity, i.e., metabolic syndrome and diabetes. And, of course, numerous studies have consistently found that coronary heart disease deaths are less likely for persons who consume moderate amounts of alcohol [17].

Our findings should not lead researchers or clinicians to conclude that high levels of alcohol consumption are healthy. In addition to the risk of alcoholism, persons who consume large amounts of alcohol are at increased risk for depressive symptoms. Since many of the subjects in our sample already have frequent mental distress, promoting moderate use of alcohol in this population would represent a misguided effort to combat obesity with adverse health ramifications analogous to recommending cigarette smoking as a weight-loss strategy.

Our study confirmed the known relationship between poor mental health and obesity. In 2001, the Behavioral Risk Factor Surveillance System (BRFSS) survey found that 10% of adults reported frequent mental distress (poor mental health ≥ 15 days per month). Persons reporting frequent mental distress (FMD) were more likely to have adverse, but modifiable, health behaviors including smoking, drinking, physical inactivity, and obesity [19]. In a study examining BRFSS data aggregated across several years, 12.3% of women reported frequent mental distress and this group was more likely to consume cigarettes and alcohol [20]. Hence, persons with frequent mental distress are more likely to have physical problems that require treatment in primary care settings and engage in behaviors that make treatment more difficult. Identifying and treating the underlying causes of mental distress is important from an economic, medical, and psychological perspective [21-25].

In our study, persons with obesity were more likely to report frequent mental distress than persons who were not obese. "Frequent mental distress" is not diagnostic of any particular psychiatric illness, such as depression. Nevertheless, the increased self report of mental distress among patients with obesity suggests that psychopathology, potentially treatable, may be unrecognized in a group with several identifiable risk factors for depression.

In July 2003, the President's New Freedom Commission published recommendations calling for enhanced efforts to screen for mental disorders in primary care settings [26]. Depression screening in primary care settings has also been recommended by the US Preventive Task Force (USPTF) [27]. Obese patients represent a readily identifiable subgroup of patients that can clearly benefit from the use of office-based screening tools that are brief, inexpensive, and easy to administer. Despite the availability of a number of instruments available to screen for mental disorders in primary care settings, such screening often does not occur [28-30]. Although the reasons why screening has not been widely implemented are unclear, they include time constraints, lack of reimbursement, stigma (on the part of both the patient and the physician), and financial barriers to effective treatments (e.g., psychotherapy and antidepressant agents).

Finally, we note that health confidence, believing that one can control one's own health, appears to have a protective effect against obesity. Earlier research has shown that health confidence, sometimes called medical skepticism, is related to better self-rated health [31].

There are several limitations to this study. The use of self-reported data for height and weight may result in underestimation of true BMI. Additionally, the study population was intentionally chosen to over sample high-risk populations and may not be representative of the general community population. Epidemiological studies have demonstrated an association between physical and psychiatric disorders. Using a convenience sample of ambulatory care patients and their families may over represent the portion of persons with poor mental health. Lastly, this survey was taken from a rather homogeneous group that was relatively young. Only 13% of persons in this study were over 65 years old. The results of this study may not be applicable to all groups, especially geriatric patients.

## Conclusion

Our findings demonstrated an association between obesity and frequency of alcohol consumption. This result is reminiscent of the discovery that moderate alcohol consumption was related to reduced risk of heart disease [17]. An accumulation of evidence on this point led some physicians to recommend consumption of one or two drinks a day to their patients. If other researchers find occasional drinking is related to reduced risk of obesity, a similar change in medical advice could occur.

The mechanism by which alcohol use might reduce obesity is not immediately clear. Some investigators have observed that consumption of sugars is greater among non-drinkers [32]. It is possible that alcohol is an appetite suppressant for regular drinkers who do not binge.

While the association between frequent mental distress and obesity is not new or surprising, recognition of that relationship may provide an easy, effective, and important screening mechanism to detect depression in primary care settings. We believe that obesity, particularly obesity in women, may identify a high risk group that can be targeted for depression screening in primary care settings. Replication of these results by other investigators is important. In particular, establishing a direct association between frequent mental distress and depression among obese women will be important in improving the health care in this group of patients.

Obesity seems to be a phenomenon of middle age. This suggests a cohort effect, probably related to community changes in energy balance in the past few decades (e.g. increasing eating quantity and calorie density, and decreasing physical activity. We also note that obesity in this sample seems to be associated with a constellation of personal characteristics: non-smoking, TV watching, mentally distressed, and alcohol abstaining. While it is tempting to try to "explain" each of these characteristics individually (e.g. smoking is known to reduce body weight), it might be more productive to look at them as a personality pattern. For example, if this pattern were interpreted as describing passive people with little self-efficacy, then it might be more effective to try changing their environments than their volitional behavior. Research projects are needed that sort out causal mechanisms and test 'health protection' approaches to weight control: i.e., experimental interventions targeted at changing the environments of the middle-aged person who may be unable to change her or his own behavior.

## Competing interests

The author(s) declare that they have no competing interests.

## Authors' contributions

JR planned the study, organized the survey and wrote the introduction, discussion and conclusions. BR wrote sections relating to mental health and AW wrote sections relating to family practice. AD analyzed the data and wrote the first draft of the methods and results.

## Pre-publication history

The pre-publication history for this paper can be accessed here:


